# Retraction of the Research Article: “Police Violence and the Health of Black Infants”

**DOI:** 10.1126/sciadv.aba5491

**Published:** 2019-12-12

I recently published a Research Article entitled: “Police Violence and the Health of Black Infants,” (*1*) in which I examined the impact of in utero exposure to police killings of unarmed blacks in a residential environment on black infants’ health. Subsequent to publication, a reader discovered classification errors in the data openly shared as part of the publication. After learning about these errors, I conducted a thorough investigation focusing on a larger sample of cases that revealed further classification errors. A reanalysis of the data leads to revised findings that do not replicate the results in the original paper. Therefore, I must retract the Research Article and apologize that these errors were not discovered before publication. I am grateful that someone found the classification errors, which allowed me to investigate the issue and correct it quickly.
